# Thyroxine and treatment of hypothyroidism: seven decades of experience

**DOI:** 10.1007/s12020-019-02006-8

**Published:** 2019-07-18

**Authors:** Roselyn Cristelle I. Mateo, James V. Hennessey

**Affiliations:** 1 Division of Endocrinology, Department of Medicine, Rush Medical College, Rush University Medical Center, Jelke Building 6th Floor, 1750 Harrison Street, Chicago, IL 60612 USA; 2 Division of Endocrinology, Diabetes and Metabolism, Department of Medicine, Beth Israel Deaconess Medical Center, Harvard Medical School, 330 Brookline Avenue, Gryzmish 6, Boston, MA 02215 USA

## Abstract

Hypothyroidism is one of the most common endocrine disorders, affecting as much as 10% of the global population. There is a rich cultural milieu of treatment history and interventions dating as far back as 2 millennia. Chinese cretins were treated with sheep thyroid in the 6th century. In 1890, transplanted animal thyroid tissue resulted in a prompt clinical response in a myxedematous patient, and in 1891 injections of sheep thyroid were reported. One year later, the oral administration of fresh sheep thyroid glands was noted to be effective. Within a few years, the danger of over-dosage with extracts was recognized and dosing guidance indicated a low dose start and gradual increase as required based on symptoms. Orally ingested extracts became widespread and by 1914 thyroxine had been crystallized. In 1927, thyroxine, was synthesized as an acid, limiting oral absorption. Finally a sodium salt of thyroxine was introduced in 1949. These synthetic preparations were then made available for clinical use. Prior to 1970, extracts and combination therapy with synthetic LT4 and LT3 were standard replacement until the peripheral deiodinase-mediated T4 to T3 conversion documented the endogenous generation of T3 from LT4 in athyreotic subjects. This resulted in advocacy for patients previously treated with combinations and desiccated thyroid be transitioned to l-thyroxine monotherapy. The determination of the optimal dose has evolved such that now a general recommendation for replacement dosage of LT4 is 1.6–1.7 mcg/kg/day. Thyroid hormone extracts were established prior to the FDA’s establishment in 1906, and when the Food, Drug, and Cosmetic act of 1938 enhanced the FDA’s regulatory authority. In 1997, FDA declared LT4 products to be new drugs subject to regulation and quickly a pharmacokinetic process to determine interchangeability among approved LT4 products ensued. Differences in bioavailability of 12.5% or more may be considered therapeutically equivalent and therefore such products interchangeable. To assure refill to refill consistency, all levothyroxine sodium products now meet a 95–105% potency specification throughout their labeled shelf-lives. Seventy years after Kendall’s great achievement in isolating thyroxine, we have thyroxine products with precise amounts of synthetic hormone that meet demanding regulations to assure high product quality, predictable bioavailability given its narrow therapeutic range, and now are left with potential variance in the therapeutic efficacy among different preparations.

Hypothyroidism is one of the most common endocrine disorders, affecting as much as 10% of the global population [1]. Unknown to many, a rich cultural milieu surrounds its treatment history with physician discoveries and interventions dating as far back as 2 millennia.

## Treatment in the pre-20th century era

As early as the 6th century, there were documented reports of Chinese cretins being treated with sheep thyroid [2]. In 1883, Kocker and Semon observed that thyroidectomy resulted in the onset of myxedema [cachexia strumipriva] based on a survey of surgeons [3]. In 1890, Bettencourt and Serrano transplanted animal thyroid tissue into a myxedematous patient that then led to a prompt clinical response, including an increase in temperature [4]. In 1891, a young English physician, George Murray, reported injecting patients with myxedema, with thyroid extract derived from sheep thyroids, but received little support from the medical community [5]. Considerable interest was apparent when he documented beneficial effects in a 46-year-old patient. His third and fourth patient died of cardiac failure, presumably myocardial infarction, however his first patient survived nearly 30 years and he continued to injected sheep extract intermittently [2, 6]. One year later, physicians MacKenzie and Fox reported the oral administration of fresh sheep thyroid glands was effective in reversing the signs and symptoms of hypothyroidism [7].

In these early reports, the clinical benefits of thyroid extract administered to clinically myxedematous patients included rapid weight loss, measurable increases in body temperature, urine volume, and nitrogen excretion [8]. Needless to say, the preparation of injectable thyroid extracts was very demanding, time consuming, and expensive [8], and as a result of acute and chronic complications associated with these injections, there was a move to transition to oral preparation of extracts [9]. Within a few years, the danger of over-dosage was recognized and dosing guidance and cautions in regard to iatrogenic over-dosage appeared, resulting in recommendations that treatment start with a low dose and gradually be increased as required. Patients were considered adequately treated by experienced physicians if they were no longer complaining of hypothyroid symptoms, and not demonstrating obvious thyrotoxic symptoms [10]. The non-specificity of “hypothyroid symptoms” is now deemed unreliable to be used as an endpoint [11–13] but objective measures of success including body weight, pulse rate, skin quality, sensitivity to cold, and quantitative measurement of Achilles tendon relaxation time were documented and accepted universally [14].

## Discoveries in the 20th century

As use of orally ingested animal thyroid gland extracts became more widespread, scientists sought to identify the vital ingredient responsible for overcoming the symptoms of hypothyroidism [15]. The year 1914 was a pivotal year, when Edward Calvin Kendall crystallized a substance containing 65.3% iodine. However, it would take almost 15 months before he would be able to obtain 33 g of this crystalline substance that would allow studies on its physiologic properties [16]. In 1927, for the first time, this substance, which would be later on called thyroxine, was synthesized by Harington and Barger, and revealed a molecular form which linked dietary iodine to the thyroid gland and provided a mechanism for the regulation of growth, differentiation, and metabolism [17]. Harington also recognized that, although thyroxine occurred naturally as a racemic mixture of the levo and dextro forms of the chemical, it was the levo form that had greater physiologic activity [18, 19]. The synthesized molecule was an acid, limiting oral absorption, and the clinical utility of the hormone was limited until a sodium salt of thyroxine was introduced in 1949. In 1952, Gross and Pitt-Rivers detected the second active thyroid hormone—triiodothyronine [20–22], which at the same time also was demonstrated by French investigators [23]. These synthetic preparations were made available for clinical use and were initially introduced into the US market without the need for approval by the United States Food and Drug Administration, FDA, as they were viewed as forms of thyroid hormone contained in the then commonly used thyroid hormone extracts that predated FDA regulation.

## The switch from combination therapy as a standard to monotherapy

Prior to 1970, based on several studies exploring alternatives to thyroid hormone extracts, combination therapy with synthetic LT4 and LT3 were accepted as standard appropriate thyroid hormone replacement therapy in order to mimic normal thyroid function [14, 24]. As synthetic levothyroxine and liothyronine cannot be distinguished from the two thyroid hormones produced naturally in the human body, they thus achieved comparable clinical results to thyroid hormone extract products [25].

There was hesitation in using LT4 monotherapy over a concern that it might result in T3 deficiency, but two major events in the 1970s led to changes in clinical practice that provided a justification of L-thyroxine monotherapy. The first was the identification of peripheral deiodinase-mediated T4 to T3 conversion and the second was the development of serum thyroid hormone and thyroid-stimulating hormone radioimmunoassays. Braverman et al. documented the endogenous generation of T3 from LT4 in athyreotic subjects [26]. Evidence emerged that T3 is predominantly produced by peripheral conversion through the 5′-deiodination of T4 and only 20% of T3 in the circulation is secreted directly by the thyroid [27]. Subsequently, Surks et al. confirmed the restoration of the prohormone pool and endogenous generation of T3 in those treated with LT4 monotherapy [28, 29], thus providing a solid mechanism to explain the documented ability of LT4 monotherapy clinically to normalize thyroid function.

Studies were conducted comparing monotherapy with combination therapy leading to the conclusion that subjects were unable to distinguish treatment with LT4 from LT4/LT3 combination treatment. Although as many as 18% preferred LT4/LT3, there was a relatively high incidence of side effects among those using the combination [24].

## Radioimmunoassay-based thyroid function tests

In 1973, Surks reported that substantial supraphysiologic peaks of T3 were noted following ingestion of desiccated thyroid extracts and liothyronine (LT3), while 24 h hormone profiles similar to euthyroid controls were observed following the chronic ingestion of levothyroxine (LT4) monotherapy [28]. The development of these radioimmunoassays provided the first sensitive and specific markers of systemic thyroid hormone action and it was observed that LT4 monotherapy could normalize both T4 and T3 at the expense of a high T4:T3 ratio [30, 31]. Preparations of LT3, DTE, and fixed dose LT4/LT3 combination typically resulted in low or low-normal serum T_4_ values with elevated postabsorptive serum T_3_ levels, and a low T_4_:T_3_ ratio resulting in a major shift in treatment of hypothyroidism [32]. Clinicians next titrated therapy by focusing efforts on the normalization of TSH as a specific tissue marker of replacement adequacy given its ease of measurement, predictability, and lack of untoward outcomes. One study looking at long-term outcomes of middle-aged women treated with LT4 monotherapy documented an average of 12 years of follow up and captured the clinical status of 99.7% of the participants. Eighty three percent of these women were still on LT4 at follow up and 92% had TSH and TT3 within the normal range. Compared with a euthyroid control group, subjects had similar laboratory and clinical data, quality of life and incidence of myocardial infarction, diabetes mellitus, cerebrovascular accidents, cancer, and death [33].

## Natural thyroid products fall out of favor

In 1965, four of every five prescriptions for thyroid hormone were for natural thyroid preparations, as pharmacologic authorities confirmed that these were highly effective, well-absorbed, and produced clinically predictable outcomes, but reports of patients not responding to desiccated thyroid raised concerns about inconsistencies in the potency of these tablets [34]. There were reports that included enhanced to no detectable metabolic activity [35] and limited shelf life of commercially available thyroid extract products especially in humid conditions [36]. In 1985, the U.S. Pharmacopeia standard for assessing thyroid hormone products shifted from determining the iodine content to directly measuring the T3 and T4 content to predict a more reliable potency, but by then natural thyroid products had fallen out of favor [35]. Some clinicians advocated for patients previously treated with desiccated thyroid be transitioned to l-thyroxine monotherapy [37–41].

## The determination of the optimal dose

From the time that LT4 was first introduced, individually tailored dosing using regulated preparations and eventually using serum TSH to determine the clinical and biochemical status of patients have also evolved. Symptom-based clinical assessments gave way to objective biochemical investigations, and clinicians recognized clear evidence of both overdosage and underdosage. Suppressed TSH or TSH response to thyrotropin releasing hormone (TRH) and/or elevated T3 were seen as markers of overreplacement [26, 42] and elevated basal TSH or exaggerated TSH response to TRH [43] when patients appeared to be “clinically euthyroid” were considered markers of under-replacement. As a result of FDA mandated changes in the quality control process of commercial products prior to release [44], the LT4 content of tablets became more uniform and predictably similar to the labeled amount [45]. This resulted in patients achieving similar control at lower mcg/kg doses of the newer generation of LT4 preparations [46]. With the aid of biochemical TSH assessment, the estimated LT4 replacement dose declined steadily from the 300+ mcg daily (>3 mcg/kg) range [24] to more moderate doses of 1.6 to 2.0 mcg/kg daily [47] and it was observed that adequate thyroxine dosing required less per kilogram body weight in the elderly [41, 48]. These findings resulted in the general recommendation for estimating full replacement dosage of LT4 to be about 1.6–1.7 mcg/kg/day [49, 50].

## Response of peripheral markers of hypothyroidism with l-thyroxine monotherapy

Soon after l-thyroxine monotherapy at doses to normalize the serum TSH became the standard of care, there remained a subgroup of patients with residual symptoms of hypothyroidism despite normalization of the serum TSH, which raised concerns of potential inadequacies of levothyroxine monotherapy and its ability to universally normalize markers of hypothyroidism especially in patients with deiodinase polymorphisms [51].

Short term studies in l-thyroxine-treated patients for 3 months who achieved a normal serum TSH observed that basal metabolic rate (BMR) remained about 10% less than that of normal controls and doses of l-thyroxine that subsequently normalized the BMR in the short term were associated with suppressed serum TSH and caused iatrogenic thyrotoxicosis [32, 52, 53]. Dyslipidemia, manifested as an elevation of low-density lipoprotein and total cholesterol levels, can also be affected by thyroid dysfunction. Although early studies demonstrated equivalent cholesterol responses while on relatively high doses of LT4 or DTE, an analysis of 18 studies on the effect of thyroid hormone replacement on total cholesterol levels in patients with overt hypothyroidism showed a reduction in the total cholesterol level in all 18 studies. However, in some studies, the mean post treatment total cholesterol level remained above the optimal range (>200 mg/dL [>5.18 mmol/L]) [54] suggesting that either lipid measures are not fully restored despite normalization of the serum TSH [55], the subjects differed in other ways from the controls or the short duration of the observations were inadequate to assure so that the full effect of LT4 was seen. The clinical significance of the degree of remaining dyslipidemia in patients treated with l-thyroxine with a normal TSH is unknown, given that the benefit of thyroid hormone replacement in subclinical hypothyroidism is itself controversial [56, 57]. Relatively low serum T_3_ levels have been suggested to contribute to residual manifestations attributed to persistent hypothyroidism, however others have speculated that the higher serum T_4_:T_3_ ratio might impair systemic T_3_ production via downregulation of a deiodinase pathway, although this has not been definitively documented [58].

Studies in patients with hypothyroidism rendered biochemically euthyroid with LT4 have demonstrated what appears to be residual impairment in health-related quality of life as assessed using self-administered ThyPRO and SF-36 questionnaires [59]. There are many physical factors such as BMI, comorbidities, additional medications, in addition to psychological, social, economic, cultural, and labeling factors that contribute to quality of life [60]. Preference for combination LT4/LT3 therapy has been associated with weight loss achieved during therapy in both a trial of synthetic combination therapy and in a trial of desiccated thyroid extract, but has not been found to be a factor by others [61]. Many patients with hypothyroidism have additional chronic conditions and the overlap in symptoms associated with these conditions and hypothyroidism such as fatigue, memory impairment, and sleep disturbance is apparent [62]. Patients with hypothyroidism are more likely to be diagnosed with disorders such as anxiety and depression [63]. An ascertainment bias thus becomes a major factor because patients who do not feel well are more likely to be screened for and diagnosed with hypothyroidism given an assumption of causation [64].

As the symptoms of hypothyroidism are very nonspecific, and being labeled with a chronic illness has a significant negative impact on self-reported health status [60], the presence of symptoms in LT4 treated patients should be expected but are likely not due to thyroid hormone deficiency when TSH is normal [65].

## FDA regulations

Thyroid hormone extracts were an established therapeutic intervention prior to the FDA’s establishment in 1906, and were the sole thyroid replacement option in use when the Federal Food, Drug, and Cosmetic act of 1938 enhanced the FDA’s regulatory authority. As LT4 and LT3 were subsequently recognized as components of thyroid hormone extract, they were not initially subjected to oversight regulation when introduced in the 1950’s. Only thyroid hormone products that were introduced after 1938 were potentially subject to this higher level of regulation, although initially the FDA declined to do so, enhanced scrutiny was eventually imposed when concerning events came to light [66–68].

Prior to 1980, the standard by which the United States Pharmacopeia (USP) assessed the potency of thyroid hormone and thyroid extract tablets was based on their iodine content [43] and products were considered to meet that standard if the iodine content fell within the prescribed limits. Each manufacturer conducted their own manufacturing processes and created formulations to maintain this iodine standard without FDA oversight [9], however this unregulated process resulted in significantly conflicting results when various preparations were compared with one another [45, 69] and even amongst different batches within the same brand [44, 70, 71].

In 1980, with the introduction of high performance liquid chromatography (HPLC), differences in LT4 tablet content, and subsequent differences in clinical outcomes were detected, despite using products meeting the standards of iodine content [45, 72]. This HPLC standard was then adopted by the USP to assure predictable LT4 content for all products distributed in the US.

In 1997, following receipt of 58 adverse drug experience reports indicating that products had failed to maintain potency through the expiration date and the amount of active ingredient of the same dosage and brand varied from lot to lot, the FDA decided that it was of utmost importance to ensure that the amount of available active drug be consistent for a given tablet strength. The FDA declared LT4 products to be new drugs subject to new drug applications (NDA) in order to be distributed in the US. A call for major change in the documentation of the manufacture, quality control, distribution, and use of LT4 products was stipulated by the FDA [49, 50]. Soon after, the NDA procedures were in place, a process of evaluating the potential interchangeability among approved LT4 products ensued. Bioequivalence (BE) has been defined as the absence of a significant difference in the rate and extent to which the active ingredient in a pharmaceutical equivalent becomes available at the site of drug action when administered in the same molar dosage [73]. BE studies are seen by the FDA as a means of evaluating the safety of substitution of one product with another at the same dosage without concern for potential adjustment in drug dose or the need for any follow up therapeutic monitoring. Pharmacokinetic studies have become a critical component of the abbreviated new drug application (ANDA) submission process intended for the approval of generic products. The BE of levothyroxine formulations is assessed by comparing pharmacokinetic measures of T4 absorption, rather than TSH or T3 levels [74]. It is understood that systemic T_4_ levels do not reflect the levels of thyroid hormone levels at the site of action, but it is assumed that a relationship exists between the efficacy and safety of the product and its systemic levels [75]. Despite TSH sensitivity to changes in thyroid hormone level, TSH is not used to assess BE of thyroid formulations because it is considered a secondary response to levothyroxine and there is a time delay between the administration of exogenous levothyroxine and the changes noted in TSH levels.

Differences in bioavailability among LT4 preparations of equivalent dosage have altered clinical outcomes when switches occur from one product to another [9, 76]. FDA BE criteria allows differences in bioavailability of 12.5% or more to be considered therapeutically equivalent and therefore interchangeable. Recognizing the narrow range within which LT4 must be titrated, the FDA has issued letters to all NDA and ANDA holders requiring that they change the specifications for their products so that all levothyroxine sodium products approved for use in humans will meet a 95–105% potency specification throughout their labeled shelf-lives, instead of previously acceptable potency limits of 90–110% [73, 77].

All of these regulations are part of the FDA effort to address concerns about variability in the stability profile of levothyroxine sodium products, reduction in fluctuations of drug concentrations, and the potential clinical consequences of this variability in achieving target thyroid hormone levels. This is a concern in particularly vulnerable patients, such as those with thyroid cancer, the elderly, and children. These regulations are also intended to ensure that levothyroxine sodium drug products maintain their quality throughout their shelf lives to ease concerns about refill to refill variance.

## Dosing considerations

When dosing LT4, body weight, gender, and age should be considered when estimating and titrating to biochemical endpoints. Consideration must be given when substituting LT4 preparations with differing bioavailability as these may result in subtle, but measurable differences in clinical and TSH outcomes. Compliance, duration of therapy, renal thyroxine loss, and the potential impact of other interfering medications or food intake also play a role and may impact efforts to achieve optimal control [78].

## Continued differences in preferences

National and international guidelines on the treatment of hypothyroidism in adults, clearly advocate use of LT4 monotherapy as the first line approach to patients with hypothyroidism. However some continue to prefer the use of thyroid hormone extracts as an alternative to LT4 alone or in combination with triiodothyronine. Initially, the use of combination therapy with synthetic LT4 and LT3 was introduced to provide a more predictable dosage, and was considered an alternative medication to thyroid extracts. This combination of LT4/LT3 has subsequently been proposed to be superior to LT4 monotherapy among patients reporting symptoms attributed to hypothyroidism. However, most studies conducted have used different ratios of LT4 to LT3 and have been unsuccessful in demonstrating superior objective outcomes [66, 79].

Guidelines differ as to when to consider the addition of LT3 therapy. The European Thyroid Association suggests the experimental use of LT4/LT3 in a 14:1 ratio and the most recent American Thyroid Association (ATA) guideline considers LT4/LT3 to be an experimental alternative as the data are insufficient to recommend its use [50].

## Future research

Seventy years after Kendall’s great achievement in isolating thyroxine, we have come a long way in the treatment of hypothyroidism: from partially purified extracts of animal thyroid gland to oral administration of precise amounts of synthetic hormone. From escaping FDA oversight to having modern LT4 subject to demanding regulations to assure high product quality, predictable bioavailability given its narrow therapeutic range, and now are left with potential variance in the therapeutic efficacy among different preparations. US-based societies such as the American Association of Clinical Endocrinologists, The Endocrine Society, and ATA have issued statements regarding concerns with the FDA’s strategies for establishing generic interchangeability using pharmacokinetic methods to establish therapeutic equivalence. Though the absolute frequency with which adverse events occur in LT4 treated patients is not clear, a survey study has identified nearly 180 incidents associated with pharmacy substitution of one product for another [80].

In regard to the generic substitution of one LT4 product for another, the three major US-based endocrine societies have released dosing, prescribing, and monitoring guidelines for those on LT4 replacement. If the brand of levothyroxine medication is changed, either from one brand to another brand, from a brand to a generic product, or from a generic product to another generic product, serum TSH should be remeasured in 6 weeks, and dose adjusted as needed. Even small changes in levothyroxine administration can cause significant changes in TSH serum concentrations, hence the need for measuring precise and accurate TSH levels to avoid potential adverse iatrogenic effects. The three societies have recommended that different methodologies to assess clinical effects and evaluation with the use of TSH, rather than pharmacokinetic studies should be explored to determine BE and true therapeutic equivalence. Until then, great attention should be put on maintaining patients on the same levothyroxine product as much as possible [50].
